# Male and female breast cancer: the two faces of the same genetic susceptibility coin

**DOI:** 10.1007/s10549-021-06159-x

**Published:** 2021-05-03

**Authors:** Susana Nunes Silva, Bruno Costa Gomes, Saudade André, Ana Félix, António Sebastião Rodrigues, José Rueff

**Affiliations:** 1 Center for Toxicogenomics and Human Health, NOVA Medical School, NMS, Universidade Nova de Lisboa, Rua Câmara Pestana, No.6, 1169-056 Lisbon, Portugal; 2 Department of Pathology, Portuguese Institute of Oncology of Lisbon, 1099-023 Lisbon, Portugal; 3 NOVA Medical School, Universidade Nova de Lisboa, 1169-056 Lisbon, Portugal

**Keywords:** Male Breast Cancer, Genetic variants in male and female breast cancer, DNA repair genes, *TP53*, *XRCC1*, *MUTYH*

## Abstract

**Background:**

Breast cancer (BC) is the most common cancer in women. In contrast, male BC is about 100 times less common than in women, being considered a rare disease. Male BC may be a distinctive subtype of BC and available data seems to indicate that male BC has a higher dependence on genetic variants than female BC. Nevertheless, the same prognostic and predictive markers are used to determine optimal management strategies for both male and female BC. Several studies have assessed the role of genetic polymorphisms (SNPs) in DNA repair genes in female BC susceptibility. However, data on male BC is scarce. Thus, the current study aimed to assess the role of SNPs in *XRCC1, MUTYH* and *TP53* genes in a male cohort of BC, and, in addition, compare the male data with matched results previously genotyped in female BC patients.

**Methods:**

The male BC cohort was genotyped through Real-Time PCR using TaqMan Assays for several SNPs previously analysed in Portuguese female BC patients.

**Results:**

The results obtained indicate significant differences in BC susceptibility between males and females for the *XRCC1* rs1799782, *MUTYH* rs3219489 and *TP53* rs1042522 and rs8064946 variants.

**Conclusions:**

In males, *XRCC1* and *TP53* variants*,* when in heterozygosity, seem to be related with lower susceptibility for BC, contrasting with higher susceptibility for a *MUTYH* variant in females. These findings may help to explain the difference in incidence of BC between the two sexes.

## Introduction

Female BC is the leading cancer among women worldwide. In the last two decades, public attention and the improvement in breast imaging platforms have had a large impact in early diagnosis and screening of breast cancer resulting in a better prognosis [1, 2]. In addition, a wealth of molecular mechanisms has led to a better understanding of the disease, resulting in more efficient treatments.

In contrast, male BC is a rare and poorly understood disease that represents about 1% of all BC cases in the Western world. However, over the last decades, the incidence has been rising [3, 4]. According to the latest estimates of the American Cancer Society, in 2020 there will be approximately 2600 new male BC cases and 520 deaths in the USA [5].

Several genetic, hormonal and lifestyle/environmental risk factors for male and female BC have been established by molecular and epidemiologic studies. Nonetheless, almost all the studies on BC and the clinical trials have been focused on women and the knowledge gained is extrapolated to manage male BC patients in the clinic setting [6].

In men, genetic predisposition appears to be an important threat to BC, with clinical implications, and a positive family history in a male family member is a strong indication for genetic counselling [7]. Male BC is more frequent in older patients and displays poorer prognosis in the elderly. *BRCA2* mutations are frequent and the risk for the occurrence of non-breast primary neoplasms is higher than for female BC, suggesting differences in the underlying genetic aetiologies of male and female BC [8–15]. Nonetheless, approximately 50–92% of familial male BC arise in breast cancer families with unknown underlying genetic mechanisms contributing to tumour predisposition [7]. Moreover, published data suggests that male breast cancer has a higher dependence on genetic constitutive features than female breast cancer [16]. These features point to a better search of genetic determinants of susceptibility in male BC.

Genetic variants in DNA repair pathways’ genes have been identified in male and female BC [7]. Base Excision Repair (BER) is one of the DNA repair pathways that mainly repairs a wide variety of non-bulky exogenous and endogenous base damage and single strand breaks in damaged DNA [17]. Genetic variants in BER genes, including *XRCC1* and *MUTYH*, have been associated with the risk of developing several types of cancer including BC. However, the association between *XRCC1* and *MUTYH* polymorphic variants with BC remains controversial [17–20].

*TP53* is a tumour suppressor gene that influences cell fate and is usually mutated in several types of cancer, BC included. Moreover, *TP53* plays an important role in DNA damage signalling, cell cycle control, chromatin remodelling and apoptosis by regulating, directly or indirectly, the transcription of genes in these pathways. *TP53* is considered a high-penetrance gene, increasing female BC risk by more than four-fold [21, 22]. Female BC incidence is very high among *TP53* mutation carriers, and the most prevalent mutations occur in triple negative tumours. Conversely, the frequency of *TP53* mutations is lower in Luminal A-like subtype [23, 24]. As male BC patients are frequently Luminal A-like subtype, mutations in *TP53* gene are also rare [25]. Nevertheless, data of male BC is scarce.

Thus, the objective of this study was to genotype *XRCC1, MUTYH* and *TP53* SNPs in a cohort of males with BC and match the results with a previously studied cohort of female BC patients, in order to identify new variants that might be involved in the predisposition to male BC and above all to check if differences in gene variants might exist between males and females with BC.

## Materials and methods

### Patients and samples collection

This study involves a cohort of male BC and, for comparison, a cohort of female BC patients according to the original study for each group of genes [17, 18, 26].

In collaboration with the Pathology Department of Portuguese Oncology Institute of Lisbon (Lisbon, Portugal), a cohort of 132 male BC patients, diagnosed and treated at the Institute between 1978 and 2018 were enrolled with patient consent. Patient data including age, family history, bilaterality, presence of non-breast primary neoplasms, distant metastasis at presentation and follow-up were obtained from clinical records. The mean and median age of male BC patients at diagnosis was 65.17 and 66 years, ranging from 31 to 87 years. Familial history of BC was recorded only in 19 patients. Six patients had bilateral carcinomas and the occurrence of non-breast primary neoplasms was identified in 27 patients. Eight patients had distant metastasis at presentation. The follow-up period ranged from 6 to 396 months. Recurrence of disease was observed in 43 patients with a mean disease-free interval of 84.3 months; 38 patients died of the disease, 5 were alive with disease and 134 had no evidence of disease. The remaining 25 died of other causes.

Formalin-fixed paraffin-embedded (FFPE) blocks were prepared for tumour tissue and also of adjacent normal tissues for all male BC patients. Histological slides were reviewed and the diagnosis confirmed and classified in accordance with the WHO Classification of Breast tumours [27]. Table 1 summarizes the clinical and pathological parameters for each cohort of BC.

**Table 1:** Characteristics of the male and female breast cancer cohorts

Feature	Male	Female
*N*	132	289
Median age, years (range)	66 (31–87)	61 (30–89)
Family history of breast carcinoma	14.4%	None*
Histological diagnosis		
Invasive breast carcinomas of no special type	93.1%	87.4%
Papillary, mucinous and lobular types	4.5%	4.9%
In situ carcinoma		
	3.8%	7.7%

*All female cases are sporadic

Subsequently, DNA was extracted for all male BC patients from FFPE blocks of normal adjacent tissue as confirmed by the pathologists. Germline DNA was extracted using the FFPE RNA/DNA Purification Plus Kit (Norgen Biotek, Thorold, ON, Canada) according to the manufacturer’s recommendations. The DNA samples were eluted in 20 µL of sterile distilled water and stored at – 20 °C until further use. For DNA quantification the Qubit 4 Fluorometer (Invitrogen, Carlsbad, CA, USA) was used.

Female BC patients were divided according to the original study for each group of genes, since the number of female patients has been enlarged over time [17, 18, 28–30].

Germline DNA from female BC patients was obtained after collection of peripheral venous blood samples from each participant. DNA was extracted as described previously [17, 18] using a commercially available kit (QIAamp® DNA mini kit; Qiagen GmbH, Hilden, Germany), according to the manufacturer’s recommendations. All samples were stored at − 20 °C until further analysis.

The study was conducted in accordance with the Declaration of Helsinki. The anonymity of all BC patients was guaranteed, all studies were conducted after acquiring the written informed consent of all the individuals involved. The BC studies were approved by the Ethics Committee (EC), for female BC by EC of Faculty of Medical Sciences and for male BC by EC of the Portuguese Oncology Institute of Lisbon (UIC/821). All samples were coded and anonymized.

### SNPs genotyping

SNPs (Table 2) were selected considering a Minor Allele Frequency (MAF) above or equal to 5% for European Caucasian population (HapMap CEU). SNPs under study belong to several regions of the gene: regulatory region, coding region or non-coding region. The *TP53* SNPs were selected concerning Tag SNPs with a correlation coefficient *r*^2^ = 0.8. By definition, a tag SNP is a genetic variant localized in a specific region of the genome showing high linkage disequilibrium which represents a specific group of SNPs defining a haplotype. It is thus possible to identify several genetic interactions without looking for every SNP individually but as a group (Table 3).

**Table 2:** Identification of genetic variants included in the study

Gene	Nucleotide change	Protein change	Variant type	dsSNP ID
*XRCC1*	C/T	p.Arg194Trp	Missense	rs1799782
	G/A	p.Arg399Gln	Missense	rs25487
*MUTYH*	G/C	p.Gln335His	Missense	rs3219489
*TP53*	G/C	p.Pro72Arg	Missense	rs1042522
	C/G	–	Intron	rs8064946
	C/T	–	Intron	rs8079544
	G/A	–	Intron	rs1625895

**Table 3:** Tag SNPs involved in this study as well as the minor allele frequency MAF. ( Adapted from https://gvs.gs.washington.edu/GVS150/index.jsp)

MAF	Tag SNP
23%	**rs1042522**
14%	**rs8064946** rs11652704
10%	**rs1625895** rs2909430
5%	**rs8079544** rs9895829

Bold represent the SNPs studied and are grouped according the corresponding tagSNP

The genotyping analysis was performed by quantitative polymerase chain reaction (qPCR) using the TaqMan SNP Genotyping Assays (ThermoFisher Scientific), except for SNPs of the *XRCC1* gene in female BC patients, which were genotyped by PCR–RFLP methodologies as described [17].

### Statistical analysis

Data analysis was performed using the Statistical Package for the Social Sciences for Windows 22.0 version (SPSS, Inc.). All genotypes were coded in order to proceed with the statistical analysis. The analysis of Hardy–Weinberg frequencies for all alleles present in patients’ populations was carried out using exact probability tests available in the SNPStat software (http://bioinfo.iconcologia.net/SNPstats) [31].

Since this is not a conclusive final study but an exploratory one on the role of selected polymorphisms in male BC compared with female BC, and the data to be obtained should be looked at as proof of concept, the Bonferroni adjustment was deemed as not necessary as it is too conservative.

Differences in genotype frequency between BC patients were evaluated by the Chi-Square (*χ*^2^) test. Logistic regression was used to estimate the differences in susceptibility of BC associated with each genotype: the susceptibility levels were calculated under the codominant model and expressed as crude odds ratios (OR) and corresponding 95% confidence intervals (CI). Results were considered significant when the corresponding two-tailed *p*-values were < 0.05. The most common homozygous genotype and male gender were considered the reference classes for such calculations.

Finally, the joint effect of multiple SNPs on BC susceptibility was estimated from application of logistic regression analysis on single SNP analysis and to all possible 2 × 2 combinations of the SNPs included in this study. Samples with one or more missing genotypes were excluded from these calculations to avoid bias due to missing data. For paired SNP analysis, the combination of the most common homozygous genotypes of each individual SNP in the control group was taken as the reference category in OR calculations. Also, paired genotypes with low frequency in the study population were pooled together. The OR were not adjusted to potential risk factors, since these might vary between genders as has been stated in bibliography.

## Results

The genotypic frequencies were determined for all groups of breast cancer patients and for all SNPs under study. The results were firstly divided by biological pathway and the SNPs evaluated individually.

Also, for the largest part of SNPs, genotype distributions were in Hardy–Weinberg equilibrium (HWE, *p* ≥ 0.05), in both populations. Significant deviations from HWE were observed solely for *MUTYH* rs3219489 (*p* = 0.017) and for *XRCC1* rs1799782 (*p* = 0.014) in the male population and for *TP53* rs1042522 in the female population (*p* = 0.029).

The main aim of our study was not to identify the magnitude of the risk but the putative differences between the male and female BC populations. The question we would like to answer can be formulated as: do the genotypic frequencies differ between both genders that share BC?

To help answer this question, we performed a logistic regression between both patient populations. Considering that males develop BC later than women and the incidence rate is much lower and BC is a rare condition in men, the analysis is limited, since various other constitutive genotypes may play a role in the incidences. To help in the statistical analysis of the differences, we considered the male population as the reference group to perform the comparison of genotype frequency distributions between males and females. Since the aim was to compare genotype frequencies, no categorical classes were considered and thus the OR values are crude and not adjusted. Table 4 shows the results obtained after logistic regression analysis, with significant differences observed for *XRCC1* rs1799782 polymorphism (*p* = 0.002) and for *MUTYH* rs3219489 polymorphism (*p* = 0.027).

**Table 4:** Genotype distribution and breast cancer susceptibility for the BER polymorphisms between males and females: *XRCC1* rs1799782, *XRCC1* rs25487, *MUTYH* rs3219489

*BER* Polymorphism	MAF		Genotype frequency		*p* value^a^	OR (95% CI)^c^
	Male	Female	Males, *n* (%)	Females, *n* (%)		
***XRCC1 rs1799782***						
C/C	T: 0.04	T: 0.09	122 (93.1)	197 (82.4)	**0.003**^**b**^	1 (Reference)
**C/T**			7 (5.3)	41 (17.2)		**3.627 (1.577**–**8.341)**^**b**^
T/T			2 (1.5)	1 (0.4)		0.310 (0.028–3.451)
***XRCC1 rs25487***						
G/G	A: 0.36	A: 0.32	54 (41.2)	111 (46.4)	0.503	1 (Reference)
G/A			59 (45.0)	103 (43.1)		0.849 (0.538–1.340)
A/A			18 (13.7)	25 (10.5)		0.676 (0.340–1.344)
***MUTYH rs3219489***						
G/G	G: 0.3	G: 0.25	66 (53.7)	158 (56.0)	**0.045**^**b**^	1 (Reference)
G/C			40 (32.5)	106 (37.6)		1.107 (0.696–1.760)
**C/C**			17 (13.8)	18 (6.4)		**0.442 (0.215**–**0.911)**^**b**^

Bold represents the statistical significant *p* value < 0.05

*MAF* minor allele frequency, *OR* odds ratio, *CI* confidence interval

^a^*p*-value *χ*^2^ test

^b^*p* < 0.05

^c^ORs and 95% CI for specific genotypes were calculated using logistic regression models

The results indicate that the heterozygous genotype for the *XRCC1* rs1799782 polymorphism in female patients is related to a higher susceptibility to breast cancer than in males [OR 3.627; 95% CI 1.577–8.341]. The homozygous variant of the *MUTYH* rs3219489 polymorphism is associated with a lower susceptibility in females than in males [OR 0.442; 95% CI 0.215–0.911].

The genotypic frequencies determined for the polymorphisms identified in the *TP53* gene are shown in Table 5. Our results revealed significant differences between both populations for *TP53* SNPs rs1042522 and rs8064946. In both cases, the presence of the heterozygous genotype appears related to a higher susceptibility in the female population [OR 1.817, 95% CI 1.034–3.192; *p* = 0.038] and [OR 2.333, 95% CI 1.190–4.577; *p* = 0.014].

**Table 5:** Genotype distribution and breast cancer susceptibility between males and females for the *TP53* polymorphisms: rs1042522; rs8064946; rs8079544 and rs1625895. Male BC patients (*n* = 132) and female BC patients (*n* = 94)

*TP53* Polymorphism	MAF		Genotype frequency		*p* value^a^	OR crude (95% CI)
	Male	Female	Males, *n* (%)	Females, *n* (%)		
rs1042522						
G/G	C: 0.21	C:0.23	74 (62.2)	47 (50.0)	0.061	1 (Reference)
G/C			39 (32.8)	45 (47.9)		**1.817 (1.034**–**3.192)**^**b**^
C/C			6 (5.0)	2 (2.1)		0.525 (0.102–2.710)
rs8064946						
C/C	G: 0.11	G: 0.19	93 (81.6)	62 (66.0)	**0.036**^**b**^	1 (Reference)
C/G			18 (15.8)	28 (29.8)		**2.333 (1.190**–**4.577)**^**b**^
G/G			3 (2.6)	4 (4.3)		2.000 (0.433–9.246)
rs8079544						
C/C	T: 0.04	T: 0.06	116 (92.1)	83 (88.3)	0.357	1 (Reference)
C/T			9 (7.1)	11 (11.7)		1.708 (0.677–4.307)
T/T			1 (0.8)	0 (0.0)		N.D
rs1625895						
G/G	A: 0.13	A: 0.14	91 (75.2)	68 (73.1)	0.934	1 (Reference)
G/A			29 (24.0)	24 (25.8)		1.108 (0.593–2.070)
A/A			1 (0.8)	1 (1.1)		1.338 (0.082–21.778)

Bold represents the statistical significant *p* value < 0.05

*OR* odds ratio, *CI* confidence interval

^a^*p*-value *χ*^2^ test

^b^*p* < 0.05

^c^ORs and 95% CI for specific genotypes were calculated using logistic regression models

In order to investigate the joint effect of multiple SNPs on breast cancer susceptibility, two-way SNPs combinations were performed (Table 6) and SNP-SNP interactions among BER polymorphisms (Table 7) and TP53 polymorphisms (Table 8). The two-way SNPs interaction was performed for all SNPs under study combining the effect of BER and TP53 genes.

**Table 6:** Two-way SNP interaction among BER genes and TP53 gene: distribution of combined genotypes between male and female populations

*BER* Polymorphism	Males, *n* (%)	Females, *n* (%)	*p* value^a^	OR (95% CI)
*Two-way SNP: BER-BER*				
***XRCC1 rs1799782–XRCC1 rs25487***				
C/C–G/G	48 (93.1)	82 (34.3)	0.071	1 (Reference)
C/C–G/A	57 (5.3)	90 (37.7)		0.924 (0.568–1.504)
C/C–A/A	17 (1.5)	25 (10.5)		0.861 (0.423–1.754)
**C/T**–**G/G**	5 (3.8)	28 (11.7)		**3.278 (1.187**–**9.055)**^**b**^
RARE combinations	4 (3.1)	14 (5.9)		2.049 (0.638–6.581)
***XRCC1 rs1799782– MUTYH rs3219489***				
C/C–C/C	64 (52.9)	105 (44.9)	0.142	1 (Reference)
C/C–C/G	39 (32.2)	80 (34.2)		1.722 (0.817–3.631)
C/C–G/G	13 (10.7)	9 (3.8)		0.459 (0.142–1.490)
C/G –C/C	1 (0.8)	24 (10.3)		1.151 (0.609–2.175)
RARE combinations	4 (3.3)	16 (6.8)		0.810 (0.452–1.448)
***XRCC1 rs25487–MUTYH rs3219489***				
G/G–G/G	30 (24.4)	56 (23.8)	0.096	1 (Reference)
G/G–G/C	14 (11.4)	45 (19.1)		1.722 (0.817–3.631)
G/G–C/C	7 (5.7)	6 (2.6)		0.459 (0.142–1.490)
G/A–G/G	27 (22.0)	58 (24.7)		1.151 (0.609–2.175)
G/A–G/C	18 (14.6)	39 (16.6)		1.161 (0.569–2.368)
**G/A**–**C/C**	10 (8.1)	6 (2.6)		**0.321 (0.106**–**0.970)**^**b**^
A/A–G/G	9 (7.3)	15 (6.4)		0.893 (0.350–2.281)
A/A–G/C	8 (6.5)	10 (4.3)		0.670 (0.239–1.876)
*Two-way SNP: BER-TP53*				
***XRCC1 rs1799782– TP53 rs8064946***				
C/C–C/C	90 (78.9)	39 (52.0)	**0.001**^**b**^	1 (Reference)
**C/C**–**C/G**	16 (14.0)	17 (22.7)		**2.452 (1.125**–**5.345)**^**b**^
**C/T**–**C/C**	3 (2.6)	8 (10.7)		**6.154 (1.550**–**24.438)**^**b**^
**C/T**–**C/G**	2 (1.8)	7 (9.3)		**8.077 (1.605**–**40.641)**^**b**^
RARE combinations	3 (2.6)	4 (5.3)		3.077 (0.657–14.401)
***XRCC1 rs1799782– TP53 8079544***				
C/C–C/C	107 (86.3)	52 (69.3)	**0.014**^**b**^	1 (Reference)
**C/T**–**C/C**	6 (4.8)	13 (17.3)		**4.458 (1.604**–**12.395)**^**b**^
C/C–C/T	9 (7.3)	7 (9.3)		1.600 (0.565–4.536)
RARE combinations	2 (1.6)	3 (4.0)		3.087 (0.500–19.042)
***XRCC1 rs1799782– TP53 rs1655895***				
C/C–G/G	88 (74.6)	44 (62.9)	**0.009**^**b**^	1 (Reference)
C/C–G/A	25 (21.2)	13 (18.6)		1.040 (0.486–2.227)
**C/T**–**G/G**	3 (2.5)	11 (15.7)		**7.333 (1.946**–**27.642)**^**b**^
RARE combinations	2 (1.7)	2 (2.9)		2.000 (0.273–14.676)
***XRCC1 rs1799782– TP53 rs1042522***				
C/C–G/G	69 (58.0)	31 (41.3)	**0.019**^**b**^	1 (Reference)
C/C–G/C	36 (30.3)	26 (34.7)		1.608 (0.832–3.107)
**C/T**–**G/G**	4 (3.4)	7 (9.3)		**3.895 (1.062**–**14.286)**^**b**^
C/C–C/C	6 (5.0)	2 (2.7)		0.742 (0.142–3.884)
**C/T**–**G/C**	2 (1.7)	8 (10.7)		**8.903 (1.786**–**44.381)**^**b**^
RARE combinations	2 (1.7)	1 (1.3)		1.113 (0.097–12.737)
***XRCC1 rs25487***–***TP53 rs8064946***				
G/G–C/C	32 (28.3)	20 (26.7)	0.081	1 (Reference)
G/A–C/C	46 (40.7)	23 (30.7)		0.800 (0.378–1.694)
G/G–C/G	10 (8.8)	13 (17.3)		2.080 (0.768–5.631)
A/A–C/C	14 (12.4)	4 (5.3)		0.457 (0.132–1.586)
G/A–C/G	6 (5.3)	9 (12.0)		2.400 (0.742–7.767)
RARE combinations	5 (4.4)	6 (8.0)		1.920 (0.517–7.128)
***XRCC1 rs25487***–***TP53 rs8079544***				
G/G–C/C	46 (37.1)	28 (37.3)	0.820	1 (Reference)
G/A–C/C	56 (45.2)	31 (41.3)		
				0.909 (0.478–1.730)
A/A–C/C	12 (9.7)	7 (9.3)		0.958 (0.337–2.722)
RARE combinations	10 (8.1)	9 (12.0)		1.479 (0.535–4.083)
***XRCC1 rs25487***–***TP53 rs1655895***				
G/G–G/G	35 (29.4)	26 (35.1)	0.401	1 (Reference)
G/A–G/G	41 (34.5)	27 (36.5)		0.886 (0.439–1.790)
A/A–G/G	14 (11.8)	3 (4.1)		0.288 (0.075–1.109)
G/G–G/A	13 (10.9)	6 (8.1)		0.621 (0.208–1.852)
G/A–G/A	12 (10.1)	7 (9.5)		0.785 (0.272–2.270)
RARE combinations	4 (3.4)	5 (6.8)		1.683 (0.411–6.887)
***XRCC1 rs25487***–***TP53 rs1042522***				
G/G–G/G	30 (25.2)	17 (22.7)	0.475	1 (Reference)
G/A–G/G	35 (29.4)	19 (25.3)		0.958 (0.424–2.167)
G/G–G/A	15 (12.6)	15 (20.0)		1.765 (0.696–4.476)
G/A–G/A	17 (14.3)	15 (20.0)		1.557 (0.624–3.885)
A/A–G/G	9 (7.6)	2 (2.7)		0.392 (0.076–2.029)
A/A–G/A	7 (5.9)	5 (6.7)		1.261 (0.346–4.592)
RARE combinations	6 (5.0)	2 (2.7)		0.588 (0.107–3.244)
***MUTYH rs3219489***–***TP53 rs8064946***				
G/G–C/C	49 (43.4)	36 (39.1)	**0.043**^**b**^	1 (Reference)
G/C–C/C	32 (28.3)	22 (23.9)		0.936 (0.468–1.871)
G/G–C/G	11 (9.7)	13 (14.1)		1.609 (0.647–4.000)
C/C–C/C	12 (10.6)	3 (3.3)		0.340 (0.089–1.295)
**G/C**–**C/G**	4 (3.5)	12 (13.0)		**4.083 (1.217**–**13.702)**^**b**^
RARE combinations	5 (4.4)	6 (6.5)		1.633 (0.462–5.772)
***MUTYH rs3219489***–***TP53 rs8079544***				
G/G–C/C	57 (48.3)	43 (46.7)	0.359	1 (Reference)
G/C–C/C	35 (29.7)	33 (35.9)		1.250 (0.673–2.321)
C/C–C/C	16 (13.6)	6 (6.5)		0.491 (0.180–1.376)
G/G–C/T	6 (5.1)	8 (8.7)		1.767 (0.571–5.472)
RARE combinations	4 (3.4)	2 (2.2)		0.663 (0.116–3.787)
***MUTYH rs3219489***–***TP53 rs1625895***				
C/C–G/G	49 (41.5)	38 (41.8)	0.691	1 (Reference)
C/G–G/G	27 (22.9)	24 (26.4)		1.146 (0.573- 2.295)
G/G–A/A	13 (11.0)	5 (5.5)		0.496 (0.163–1.512)
C/C–G/A	13 (11.0)	12 (13.2)		1.190 (0.488–2.903)
C/G–G/A	11 (9.3)	10 (11.0)		1.172 (0.451–3.048)
RARE combinations	5 (4.2)	2 (2.2)		0.516 (0.095–2.806)
***MUTYH rs3219489***–***TP53 rs1042522***				
G/G–G/G	39 (33.6)	26 (28.3)	0.155	1 (Reference)
G/C–G/G	22 (19.0)	17 (18.5)		1.159 (0.519–2.591)
G/G–G/C	19 (16.4)	24 (26.1)		1.895 (0.869–4.134)
G/C–G/C	14 (12.1)	17 (18.5)		1.821 (0.768–4.322)
C/C–G/G	12 (10.3)	4 (4.3)		0.500 (0.145–1.720)
RARE combinations	10 (8.6	4 (4.3)		0.600 (0.170–2.118)

Bold represents the statistical significant *p* value < 0.05

*MAF* minor allele frequency, *OR* odds ratio, *CI* confidence interval

^a^*p*-value *χ*^2^ test

^b^*p* < 0.05

**Table 7:** SNP-SNP interaction among BER genes: distribution of combined genotypes in enrolled populations

*BER* Polymorphism	Males, *n* (%)	Females, *n* (%)	*p* value^a^	OR crude (95% CI)
***XRCC1 rs1799782***–***XRCC1 rs25487***–***MUTYH rs3219489***				
C/C–G/G–G/G	29 (25.9)	38 (16.5)	**0.008**^**b**^	1 (Reference)
C/C–G/G–G/C	13 (11.6)	37 (16.0)		2.172 (0.980–4.812)
C/C–G/A–G/C	18 (16.1)	33 (14.3)		1.399 (0.661–2.964)
C/C–G/A–G/G	27 (23.2)	52 (22.5)		1.470 (0.752–2.874)
C/C–G/A–C/C	9 (7,4)	5 (2.1)		0.424 (0.128–1.401)
C/C–A/A–G/C	8 (6.6)	10 (4.3)		0.954 (0.335–2.720)
C/C –A/A–G/G	8 (6.6)	15 (6.4)		1.431 (0.534–3.831)
**C/T**–**G/G**–**G/G**	1 (0.8)	18 (7.7)		**13.737 (1.732**–**108.955)**^**b**^
RARE combinations	9 (7.4)	26 (11.1)		2.205 (0.897–5.417)

Bold represents the statistical significant *p* value < 0.05

*MAF* minor allele frequency, *OR* odds ratio, *CI* confidence interval

^a^*p*-value *χ*^2^ test

^b^*p* < 0.05

^c^ORs and 95% CI for specific genotypes were calculated using logistic regression models

**Table 8:** SNP-SNP interaction among TP53 gene polymorphisms: distribution of combined genotypes in enrolled populations

*TP53* Polymorphism	Males, *n* (%)	Females, *n* (%)	*p* value^a^	OR crude (95% CI)
**rs1042522**–**rs8064946**–**rs8079544**–**rs1625895**				
G/G–C/C–C/C–G/G	58 (52.7)	38 (41.3)	0.145	1 (Reference)
G/C–C/C–C/C–G/G	8 (7.3)	7 (7.6)		1.336 (0.447–3.987)
G/G–C/C–C/T–G/A	17 (15.5)	14 (15.2)		1.257 (0.555–2.846)
**G/C**–**C/C**–**C/T**–**G/A**	3 (2.7)	10 (10.9)		**5.088 (1.314**–**19.694)**
G/C–C/C–C/C–G/A	6 (5.5)	8 (8.7)		2.035 (0.654–6.330)
G/G–C/G–C/C–G/A	5 (4.5)	8 (8.7)		2.442 (0.743–8.026)
RARE combinations	13 (11.8)	7 (7.6)		0.822 (0.301–2.247)

Bold represents the statistical significant *p* value < 0.05

*MAF* minor allele frequency, *OR* odds ratio, *CI* confidence interval

^a^*p*-value *χ*^2^ test

To perform the different combinations the most frequent interactions and the less frequent were pooled together and classified as “RARE combinations”. As depicted in Table 6, the two-way SNP interaction performed for polymorphisms in BER genes indicated that female patients carrying both SNPs in the *XRCC1* gene combined as heterozygous for rs1799782 (C/T) and homozygous for rs25487 (G/G) presented a higher susceptibility [OR 3.278; 95% CI 1.187–9.055; *p* = 0.022] to develop the disease.

Furthermore, the combination of *XRCC1* rs25487 and *MUTYH* rs3219489 revealed a significant correlation for the combination between the heterozygous and homozygous variant (G/A–C/C), showing a lower susceptibility for carriers of this combination in our cohort of male patients whereas women carrying the same combination [OR 0.321; 95% CI 0.106–0.970; *p* = 0.044] have a higher susceptibility.

The two-way SNP interactions between BER and *TP53* polymorphisms (Table 6) for this interaction emphasize the results of the single analyses. Overall, the significant correlations identified showed, without exception, a higher susceptibility of female gender for developing breast cancer than male gender. The correlation between *XRCC1* rs1799782 and *TP53* rs8064946 polymorphisms showed for all combined genotypes a higher susceptibility: C/C–C/G [OR 2.452; 95% CI 1.125–5.345; *p* = 0.024]; C/T–C/C [OR 6.154, 95% CI 1.550–24.438; *p* = 0.010] and C/T–C/G [OR 8.077, 95% CI 1.605–40.641; *p* = 0.011]. As mentioned above, those SNPs when analysed individually also showed the same effect as when the heterozygous genotype was present (Table 5 and Table 6). For other combinations the higher susceptibility effect was found when *XRCC1* rs1799782 polymorphism was combined with: *TP53* rs8079544 (C/T–C/C) [OR 4.458, 95% CI 1.604–12.395; *p* = 0.004]; *TP53* rs1655895 (C/T–G/G) [OR 7.333, 95% CI 1.946–27.642]; *p* = 0.003]; *TP53* rs1042522 (C/T–G/G and C/T–G/C) [OR 3.895, 95% CI 1.062–14.286; *p* = 0.040] and [OR 8.903, 95% CI (1.786–44.381); *p* = 0.008], respectively.

Furthermore, the two-way SNP interaction also presented a higher susceptibility genotype with the combination between *MUTYH* rs3219489–*TP53* rs8064946 (G/C–C/G) [OR 4.083, 95% CI 1.217–13.702; *p* = 0.023] (Table 6).

After the two-way interaction and with the purpose of investigating the joint effect of multiple SNPs, the polymorphisms were grouped together, and the susceptibility level was evaluated as shown in Tables 7 and 8. The combined genotypes’ approach was performed in two groups: one including all BER polymorphisms and the second one with selected SNPs from the *TP53* gene, avoiding very small groups of samples which might contribute to increase a bias in the results. Combining all possible genotypes for SNPs in BER genes one combination revealed a significantly higher susceptibility (Table 7). Our results showed that female patients carrying the combination between *XRCC1* rs1799782–*XRCC1* rs25487–*MUTYH* rs3219489 (C/T–G/G–G/G, respectively) have a higher susceptibility than male patients carrying the same combined genotypes [OR 13.737; 95% CI 1.732–108.955; *p* = 0.013]. The same effect was described in combined genotypes for *TP53* rs1042522–rs8064946–rs8079544–rs1625895 SNPs (G/C–C/C–C/T–G/A, respectively) [OR 5.088, 95% CI 1.314–19.694; *p* = 0.018].

## Discussion

This study compared genotype frequencies and combination of *XRCC1*, *MUTYH* and *TP53* alleles in Portuguese male BC *versus* female BC cancer patients. The sample encompassed a previously studied female BC cohort and a new cohort of 132 male BC cases in a population diagnosed and followed during 40 years in the Portuguese Institute of Oncology of Lisbon. A previous report of this male cohort indicated that male BC has specific biological characteristics [8]. However, due to the rarity of male BC, therapeutic strategies essentially follow those of female BC, not tailoring the therapy with the specificity of male BC. To achieve a personalized approach of male BC, a better knowledge of their genetic characteristics is required.

The most frequent use of Next-Generation Sequencing (NGS) has identified an increasing number of genes suspected to be involved in cancer predisposition, especially for cancers with a familial component such as BC [32]. The current use of multigene panel testing for breast cancer predisposition has been a remarkable tool, although the genes included were based on female studies, limiting its use in male breast cancer [4] and creating a partiality in the genetic predisposition analysis. More than 100 common genetic variants (SNPs) associated with female BC have been identified via genome-wide association studies (GWAS) in the general population. However, few male BC susceptibility SNPs have been identified to date.

Considering the significant difference in incidence of male vs. female BC, one cannot dismiss different SNPs frequencies in male and female BC patients. Previous studies have highlighted the role of several genes in male BC such as *BRCA2, PALB2*, *CHEK2* and *MUTYH* [4, 19, 33–36]. The majority of variants identified are included in high to moderate penetrance genes, pointing to a high incidence of familial history. GWAS studies with male BC identified SNPs that conferred greater risks of breast cancer in men than in women, suggesting a greater contribution of genetic variants to male BC than female BC predisposition [15, 16]. Some of the genes so far identified are DNA repair-related genes, which emphasize the relevance of the current study analysing genetic variants in other genes of repair pathways.

When we compared in this study the frequencies of SNPs in some BER genes and in the *TP53* gene in a cohort of male BC with previously genotyped female BC cases [17, 18, 26], the results indicate significant differences between female and male populations for the *XRCC1* rs1799782, *MUTYH* rs3219489 and *TP53* SNPs rs1042522 and rs8064946, suggesting that these genotypes are related with lower susceptibility in males for *XRCC1* and *TP53* when in heterozygosity, contrasting with high susceptibility for *MUTYH.*

To our knowledge, this is the first comprehensive study that refers the possible role of *XRCC1* and *TP53* polymorphisms in male BC susceptibility but not of *MUTYH* [4, 19]. The allelic frequency described by Rizzolo and colleagues in an Italian population (77%) is slightly different from ours in the Portuguese population (70%). In fact, the most evident difference is the frequency of the homozygous variant allele, higher in Portuguese male BC patients, while the Italian frequency is similar to our Portuguese female BC. Nonetheless, the study from Rizzolo et al. included several variants present in the *MUTYH* gene suggesting that the pathogenic variants identified might have a potential role in male BC in particular the rs34612342 variant linked to familial predisposition [19].

The combined genotypes analysis performed also intended to illustrate the biologic interaction between SNPs. Indeed, our results proved that the combination might produce an additive effect on susceptibility to BC in men. This result was more evident when *XRCC1* rs1799782–*TP53* rs8064946 SNPs were combined as shown in Table 6, as this combination might result in simultaneous DNA repair and apoptosis misfunction pointing to a potential new molecular phenotype in breast cancer susceptibility.

The main effects of SNPs in *TP53* may not necessarily directly influence cell cycle and apoptosis but may act on cell proliferation through interaction with other proteins in the p53 pathway, namely MDM2, ATM, MDM4, p21 among others [37].

The present study involved a 40-year series involving 132 patients, which allowed the double advantage of using FPPE to review each slide and also extract germline DNA for genotyping from the healthy non-tumoural margins of the tumor*.* Despite the concept of field cancerization, several studies provide evidence to justify the use of normal tissue adjacent to breast cancer tissue from paraffin-embedded tumour blocks for genotyping [38].

Indeed, regarding the use of FFPE tissue, previous publications have assessed genotype concordance between germline DNA of normal cells and DNA isolated from tissues stored in a variety of conditions and excellent genotyping concordance has been documented comparing germline DNA and DNA isolated from formalin-fixed, paraffin-embedded (FFPE) non-tumoral tissue, showing that both sources have the same germline DNA [39, 40]. Because FFPE tissues may be stored almost indefinitely at room temperature, and both DNA and RNA may be recovered from them for a significant time after fixation, these samples provide a key resource for researchers. Pathological collections preserved in paraffin are one of the richest and irreplaceable resources for the study not only of rare situations, as was the case here, but of comparative incidence.

The present study infers the existence of differences in genetic susceptibility to BC between both sexes on the basis of the possible role of *XRCC1, MUTYH* and *TP53* polymorphisms. Even taking into account differences in clinical and pathological characteristics in female and male BC, the central role played by these proteins in the control of cell proliferation, apoptosis and DNA repair may help understand the etiological basis of BC in both genders.

## Conclusions

In males, when in heterozygosity, *XRCC1* and *TP53* variants seem to be related with lower susceptibility for BC, contrasting with high susceptibility for *MUTYH*. Overall, the differences found in *XRCC1*, *MUTYH* and *TP53* polymorphisms may contribute to explain the significant difference in incidence of BC between the two sexes. Thus, female and male breast cancer may be the two different faces of a coin, but they can behave as a two-faced Janus looking at different genetic horizons.
